# Alcohol abuse in trans and sexual minority women

**DOI:** 10.1192/j.eurpsy.2022.2108

**Published:** 2022-09-01

**Authors:** P.A. Gouveia

**Affiliations:** Local health Unit of Lower Alentejo, Psychiatry, Beja, Portugal

**Keywords:** alcohol, women, female, LGBT

## Abstract

**Introduction:**

Sexual orientation is known to have some influence in alcohol consumption patterns and outcomes. Sexual minority women (SMW) are more likely to develop binge drinking and alcohol use disorder than heterossexual women. Because women tend to be more secretive with their drinking and hesitant to get help, and alcohol is particularly harmful to the female body, SMW pose particular challenges.

**Objectives:**

Review the latest research on alcoholism in SMW, focusing on various specificities of this population.

**Methods:**

Literature review in the PubMed database, using the MESH terms “alcohol use disorder”, “women”, “female”, “trans woman”, “adult”. Free full text, publication in the last 5 years, English or Portuguese and article typology filters were applied. Following primary hits, secondary references were checked and a total of 10 articles were included. Results were grouped in epidemiological, etiological, therapeutical and prognostic specificities.

**Results:**

Data indicate that lesbian and bisexual women, compared to heterosexuals, are twice as likely to engage in binge drinking. AUD prevalence and patterns of alcohol use are especially higher among younger SMW. The influence of sexual orientation on alcohol use and related outcomes seems to be greater among women than men. Regarding etiology, internalized stigma, minority stress, victimization experiences, social norms and policies are the main culprits Even though SMW are more likely to report and ask hor help, many do not receive adequate treatment. SMW show an incresed risk of developing other complications, such as physical injuries, sexual assault, liver or cardiac disease.

**Conclusions:**

Sexual minority women are particularly vulnerable to alcohol-related harms. Interventions especially directed to SMW need to be developed.

**Disclosure:**

No significant relationships.

